# Evaluating the accuracy and reliability of non-piezo portable ultrasound devices in postpartum care

**DOI:** 10.1007/s00404-024-07744-3

**Published:** 2024-10-04

**Authors:** Ruben Plöger, Charlotte Behning, Adeline Walter, Agnes Wittek, Ulrich Gembruch, Brigitte Strizek, Florian Recker

**Affiliations:** 1 Department of Obstetrics and Prenatal Medicine, University Hospital Bonn, Venusberg Campus 1, 53127 Bonn, Germany; 2 Institute for Medical Biometry, Informatics and Epidemiology, University Hospital Bonn, Venusberg Campus 1, 53127 Bonn, Germany

**Keywords:** Atony, Chip-based, Gynecology, Handheld ultrasound, Obstetrics, Puerperium, Piezo-based, Placental residues, POCUS, Point-of-care ultrasound, Postpartum complications, Semiconductors, Uterus

## Abstract

**Introduction:**

The early diagnosis of hemorrhage via postpartum ultrasound is crucial to initiate therapy and, thus, prevent maternal death. In these critical situations rapid availability and simple transport of ultrasound devices is vital, paving the way for a  new generation of portable handheld ultrasound devices (PUD) consisting of transducers and tablets or smart phones. However, evidence to confirm the diagnostic accuracy of these new devices is still scarce.

**Methods:**

The accuracy and reliability of these new devices in relation to established standard ultrasound devices is analyses in this pilot study by comparing diagnoses and by applying statistical analysis via Bland–Altman plots, intraclass correlation coefficients (ICC), and Pearson correlation coefficients (PCC). One hundred patients of a university hospital were included in this study.

**Results:**

In all cases, the same diagnosis was made regardless of the applied ultrasound device, confirming high accuracy. There was a high correlation (PCC 0.951) and excellent agreement (ICC 0.974) in the assessment of the cavum, while the assessment of the diameters of the uterus showed only a good correlation and a good agreement. Subgroup analysis for maternal weight, mode of delivery and day after delivery was performed

**Conclusion:**

The same diagnosis independent of the used devices and excellent results of the cavum assessment promote the use of PUDs in a clinical setting. The slightly lower accuracy in the measurement of the uterus may be caused by the PUD’s small acoustic window, reflecting one of its weaknesses. Therefore, the patient may benefit from the short time to diagnosis and the unbound location of examination, either in the delivery room, on the ward, or at home.

## What does this study add to the clinical work?

**Table:** 

This study was conducted to evaluate the potential of new handheld ultrasound devices in improving patient care through uncomplicated point-of-care examinations, particularly in obstetrics where rapid and accurate diagnoses in postpartum emergency situations are critical. Until now, the accuracy of these new devices in postpartum ultrasound had not been assessed.
The key findings of the study reveal that the assessment of the Cavum with these handheld devices provides diagnoses that are consistent with those from traditional ultrasound, showing near-perfect correlation and agreement. Although the correlation and agreement in measuring the uterus's diameters were slightly less accurate, they were still adequate. This minor discrepancy may be attributed to the smaller ultrasound window of the new handheld devices. Overall, the results demonstrate the accuracy and reliability of these new tools, supporting their quick and widespread use in postpartum ultrasound examinations.
This study adds to existing knowledge by extending the analysis of these new ultrasound devices, which had previously been limited to fetal biometry, to the realm of postpartum care. The study highlights the benefits of these devices in terms of flexibility and immediate use, as seen in other medical specialties, and provides evidence of their high reliability, thereby supporting their safe integration into daily clinical practice.

## Introduction

The new generation of portable handheld ultrasound devices (PUD) has become firmly established as a diagnostic tool in the fields of several medical specialties [1]. Based on chip technology, these ultrasound devices consist of transducers connected to a smart phone or tablet and, thus, are “pocket-sized” in contrast to standard ultrasound devices (SUD). In SUDs, the transducers, based on the piezoelectric effect, are connected to an ultrasound machine with a screen. In emergency situations [2] or in situations without simple access to a SUD [3], point-of-care diagnostics using the PUD are known to achieve shorter examination times than the examination with a SUD [4], so that patients benefit from a quick diagnosis and rapid initiation of treatment. However, the accuracy of the PUD in comparison to the SUD has become the subject of research in a wide range of clinical settings. These reliability studies performed in urology [5] and in internal medicine [6] indicate a good accuracy of the diagnoses made with the PUD in comparison to the SUD. In obstetrics, a highly powered study including a diverse population from the USA and Zambia [7] supported the results of pilot studies [3, 7–10] that both devices show an excellent agreement for fetal biometry. Other applications in obstetrics and gynecology [11] lack comparable data ensuring a secure use in daily clinical routines.

Postpartum ultrasound examinations could greatly benefit from the use of PUDs, through the short preparation time and the flexible point-of-care examination in any location using the small and easily transportable device. Postpartum ultrasound examinations are necessary to analyze postpartum complications such as postpartum urinary retention [12] and bleeding due to atony or placental remnants, infection or lochiostasis [13]. In the case of atony, the uterine cavity is enlarged and filled with fluid showing a mixed echo pattern. Placental remnants are presented sonographically as hyperechogenic material inside the cavity with varying blood supply, recognizable by Doppler examination [14, 15]. Before directly applying PUDs in situations with clinical pathologies, the reliability of a postpartum ultrasound examination between PUD and SUD has to be analyzed. The diagnosis, the diameter of the uterus, and of the uterine cavity were analyzed in 100 postpartum patients without clinical pathologies and, thus, likely normal postpartum uteruses using Pearson correlation coefficient (PCC), intraclass correlation coefficient (ICC), and Bland–Altman plots. Prior to the statistical analysis of the results, the examiners were asked to give feedback on their experience using the PUD. The results revealed that the same diagnosis was made regardless of the device used. Furthermore, there was a high correlation and agreement regarding the cavum measurement and a correlation and at least good agreement for the uterus measurement, so that the results regarding the reliability of his pilot study allow further studies to provide the data for a secure application of PUD in postpartum care.

## Methods

**Fig. 1:**
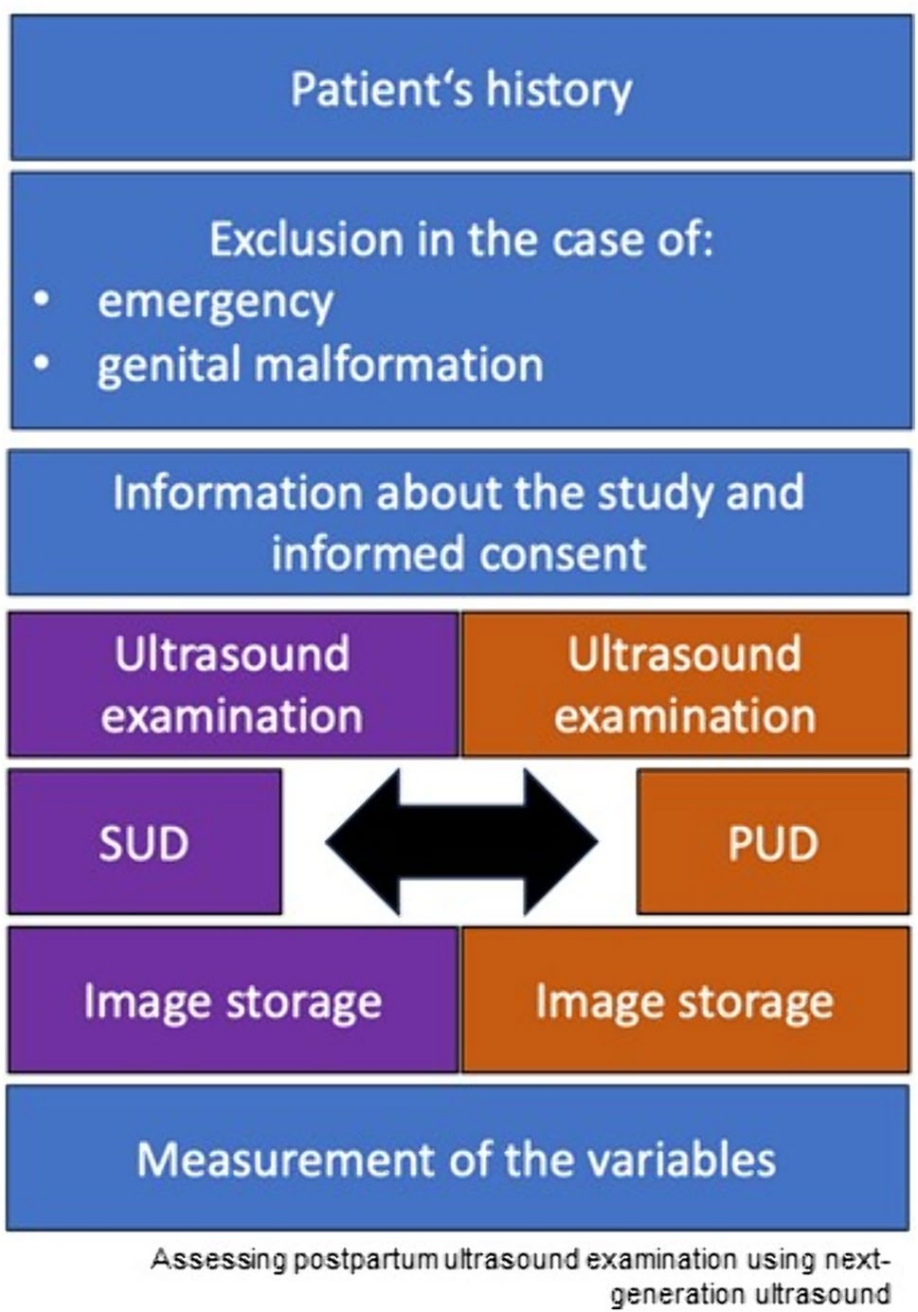
Flow chart of the study design: SUD: standard ultrasound device, PUD: portable ultrasound device

**Fig. 2:**
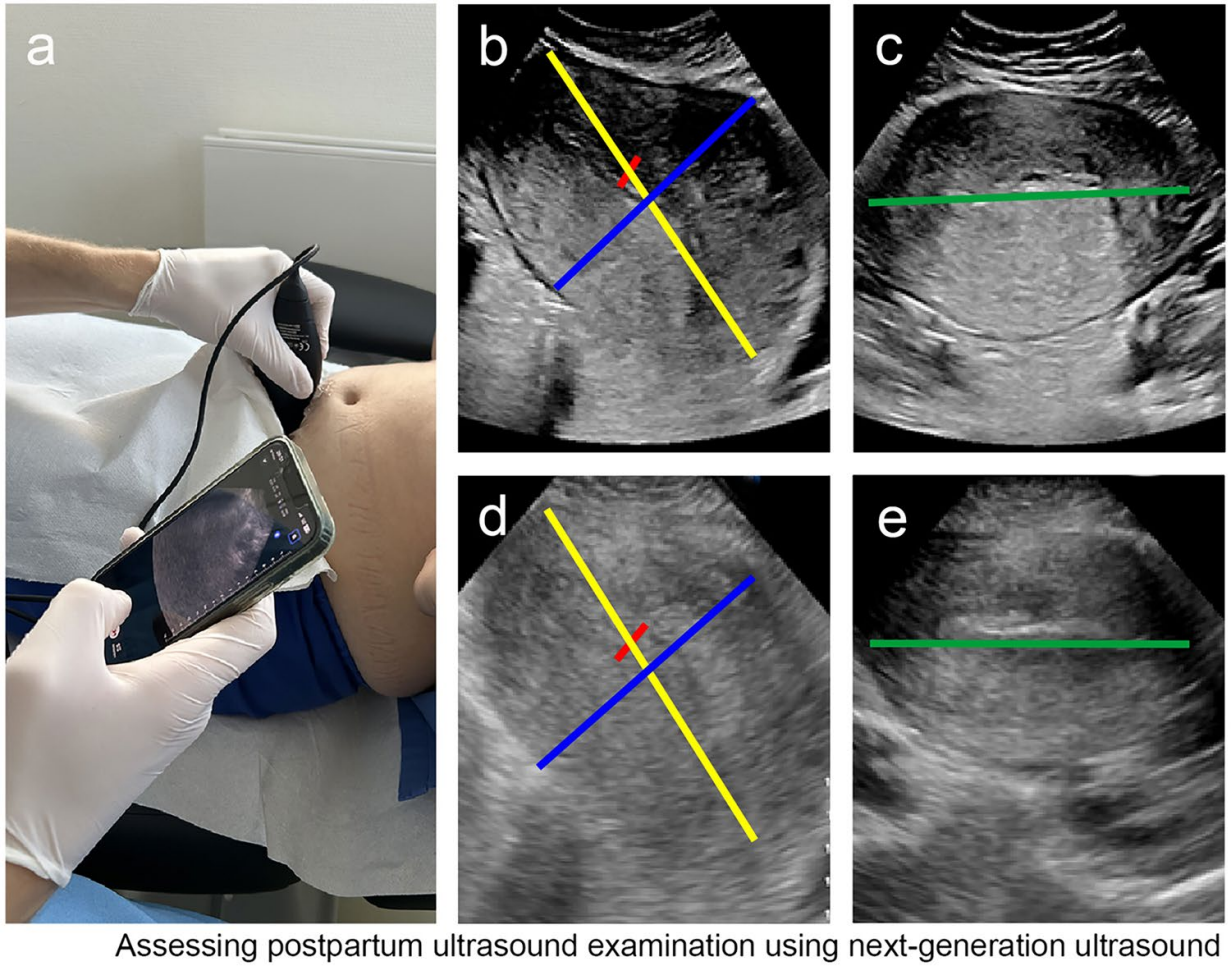
Images of an examination with a portal ultrasound machine (**a**) of transabdominal ultrasound scans using a standard ultrasound machine based on piezo technology (**b**–**c**), and of a portable ultrasound machine based on silicon chips (**d**–**e**): demonstration of the sagittal scan plan (**b**, **d**) showing the sagittal diameter (SA, yellow), the longitudinal diameter (LU, blue), the diameter of the cavum (LC, red), and of the transversal scan plan (**c**, **e**) showing the transversal diameter (HU, green)

This pilot study in form of a prospective, observational investigation was conducted over a 4-month interval (August to November 2023) within the Department of Obstetrics at the tertiary-level university hospital Bonn (Germany). The local University Hospital Ethics Board approved this study (No. 345/21). Patients upon transfer to the postnatal ward and, thus, mostly between their first and third day after delivery were the possible participants. In the case of maternal or infant complications such as neonatal hyperbilirubinemia or puerperal mastitis, the patients’ treatment caused an elongated or a subsequent stay so that the inclusion of patients several days postpartum was possible. Emergency patients and patients with genital tract malformations were excluded (Fig. 1). If a clinical pathology was identified, the patient was examined using a SUD to allow a quick diagnosis and treatment. Therefore, pathological findings in the examined population are unlikely. A further prerequisite was the operational capacity of the Department of Obstetrics to facilitate precise dual-ultrasound device examinations. The patients’ medical histories were either acquired during their first presentation in the delivery room prior to delivery or acquired by the ward physicians upon admission to the postnatal ward, in the case of puerperal complications. During daily rounds, the patients were informed about the study’s objectives and methodology. After informed consent, the participants were asked to micturate. The investigative protocol entailed the random order of the devices, whereby an image of every variable was captured using both the PUD and the SUD. The order of the variables was determined to mitigate memory bias. The same examiner conducted a unique examination on every participant with a SUD (EPIQ 5W by Philips, Amsterdam, Netherland) based on the piezoelectric crystal transducer and a PUD (Butterfly iQ by Butterfly Network, Guilford, Connecticut, USA) based on a silicon chip using the conversion of voltage to resonance (Fig. 2). Either assistant physicians (RP, AW), trained in obstetrical ultrasound for 2 years, or attendings, trained in obstetrical ultrasound for more than 2 and less than 10 years (AW, FR), were randomly assigned out of a pool of four examiners and performed the examinations. The patients’ files were used to generate the list of demographic information stored on the university hospital’s server using Excel software (Microsoft Corp., Redmond, WA, USA). The study’s primary endpoint was the agreement regarding the diagnosis. Secondary endpoints were the diameters of the uterus (horizontal axis of the uterus (HU), sagittal axis of the uterus (SU), longitudinal axis of the uterus (LU)) and the anterior–posterior diameter of the cavity (longitudinal axis of the cavity (LC)). For each variable, the examiners were asked to determine the plane with the largest diameter, subjectively without measuring, and save an image. The variables were measured after all images were taken with each device, ensuring a consistent interval between measurements of the same variable (at least 10 min). Cases with incomplete data were excluded (*n* = 2), and the study concluded upon recruiting 102 patients, so that ultimately 100 patients were included.

### Statistical methods

This pilot study included 100 participants, the number determined based on clinical feasibility and comparable studies [9]. The results of this pilot study are descriptive [16]. Statistical analyses and graphical representations were conducted using the Statistical Package for the Social Sciences (SPSS) software, version 27 (IBM Corp., Armonk, NY, USA). To evaluate the consistency and reliability of measurements derived via SUD and PUD, Pearson correlation coefficient (PCC) with a 95% confidence interval using Wald methods, intraclass correlation coefficient (ICC) employing a two-way random-effect, agreement model with a 95% confidence interval, and Bland–Altman plots [17] were performed. Before calculation of the PCC, the distribution had to be analyzed. A high degree of agreement or correlation is indicated by an ICC and a PCC approaching 1.0, as supported by the literature [18–20]. ICC is interpreted as poor by a value under 0.5, moderate by a value between 0.5. and 0.75, good by a value between 0.75 and 0.9, and excellent by value above 0.9 [20]. The mean relative difference (MRD) was calculated by taking the absolute difference between individual measurements and the aggregate mean of these measurements, summing these differences for all subjects, and then dividing by the total number of cases. Subgroup analysis based on mode of delivery, body mass index, and day postpartum was performed. The examiners were asked to give positive and negative feedback by completing a questionnaire with open questions.

## Results

Postpartum ultrasound examinations with a SUD and a PUD were performed from August to November 2023. The demographic characteristics of the study participants are outlined as follows (Table 1). Age of the participants was between 18 and 45 and the mean age was 32 years and 10 months. Many of the participants had born their first child (56%). Of the 44% multiparous participants, 72% had born their second child, 23% had born their third child, and 5% had born their fourth child. 57% of patients had a spontaneous vaginal delivery, 15% required a vacuum extraction, and 28% delivered via cesarean section. The cause of cesarean section was elective in 39% and emergent in 61%. Preconceptional body mass index (BMI) varied from 16.9 to 37.9 kg/m^2^. Analysis of BMI distribution revealed that a majority, 62%, had a BMI within the normal range (18.5 to < 25 kg/m^2^), whereas 4% were categorized as underweight (BMI < 18.5 kg/m^2^). Notably, 34% of the cohort was identified as overweight (BMI ≥ 25 kg/m^2^); within this group, 53% had a BMI between 25 and < 30 kg/m^2^, 38% were categorized as obesity class 1 (BMI ≥ 30 to < 35 kg/m^2^) and 8% as obesity class 2 (BMI ≥ 35 to < 40 kg/m^2^). 43%, 33%, 12%, 3%, 4%, 1%, and 2% of the scans were performed on day 1, 2, 3, 4, 5, 6, and 7 postpartum, respectively. The mean diameter of the HU, SU, LU, and LC were 11.63, 12.70, 6.7, and 0.67, respectively (Table 2).

**Table 1:** Patients’ characteristics

Patients’ characteristics	*n* = 100
Maternal age (year)^a^	32.8 (5.02)
Gravidity^b^	2 (1–12)
Parity^b^	1 (1–4)
Primiparous^c^	56
Multiparous^c^	44
Gestational age at delivery (week)^b^	39 + 3 (30 + 3–41 + 5)
Spontaneous vaginal delivery^c^	57
Vacuum-assisted vaginal delivery^c^	15
Cesarean delivery^c^	28
Elective cesarean delivery^c^	11
Emergency cesarean delivery^c^	17
Body mass index (kg/m^2^)^a^	24.7 (4.41)
Underweight^c^	4
Normal weight^c^	62
**Overweight and obesity**^c^	34
Overweight^c^	18
Obesity class 1^c^	13
Obesity class 2^c^	3
Obesity class 3^c^	0

^a^Mean (± standard deviation)

^b^Median (range)

^c^Number

**Table 2:** Average difference, Bland–Altman 95% limits of agreement, intraclass correlation coefficient (ICC, 95% confidence interval) and Pearson correlation coefficient (PCC with a 95% confidence interval using Wald methods) and the mean relative difference (MRD) of horizontal axis of the uteri (HU), sagittal axis of the uteri (SU), longitudinal axis of the uteri (LU) and of the cavity (LC) between the devices: standard ultrasound device using piezo technique and portable ultrasound device using silicon chip technique

	Average difference in cm	95% limits of agreement in cm	ICC (95%)	PCC (95%)	MRD (%)
HU	0.192	− 2.030 to 2.415	0.780 (0.673–0.853)	0.690 (0.545–0.835)	7
SU	0.621	− 2.933 to 4.174	0.840 (0.743–0.989)	0.783 (0.661–0.913)	9
LU	0.332	− 1.035 to 1.698	0.905 (0.820–0.945)	0.861 (0.759–0.963)	8
LC	− 0.022	− 0.254 to 0.210	0.974 (0.962–0.983)	0.951 (0.890–1.013	14

Assessing postpartum ultrasound examination using next-generation ultrasound

The determined diagnoses after the postpartum ultrasound examination using a SUD and PUD were identical. The results of the examination of the two methods show the same result. Therefore, the accuracy of the two methods is 100 percent.

**Fig. 3:**
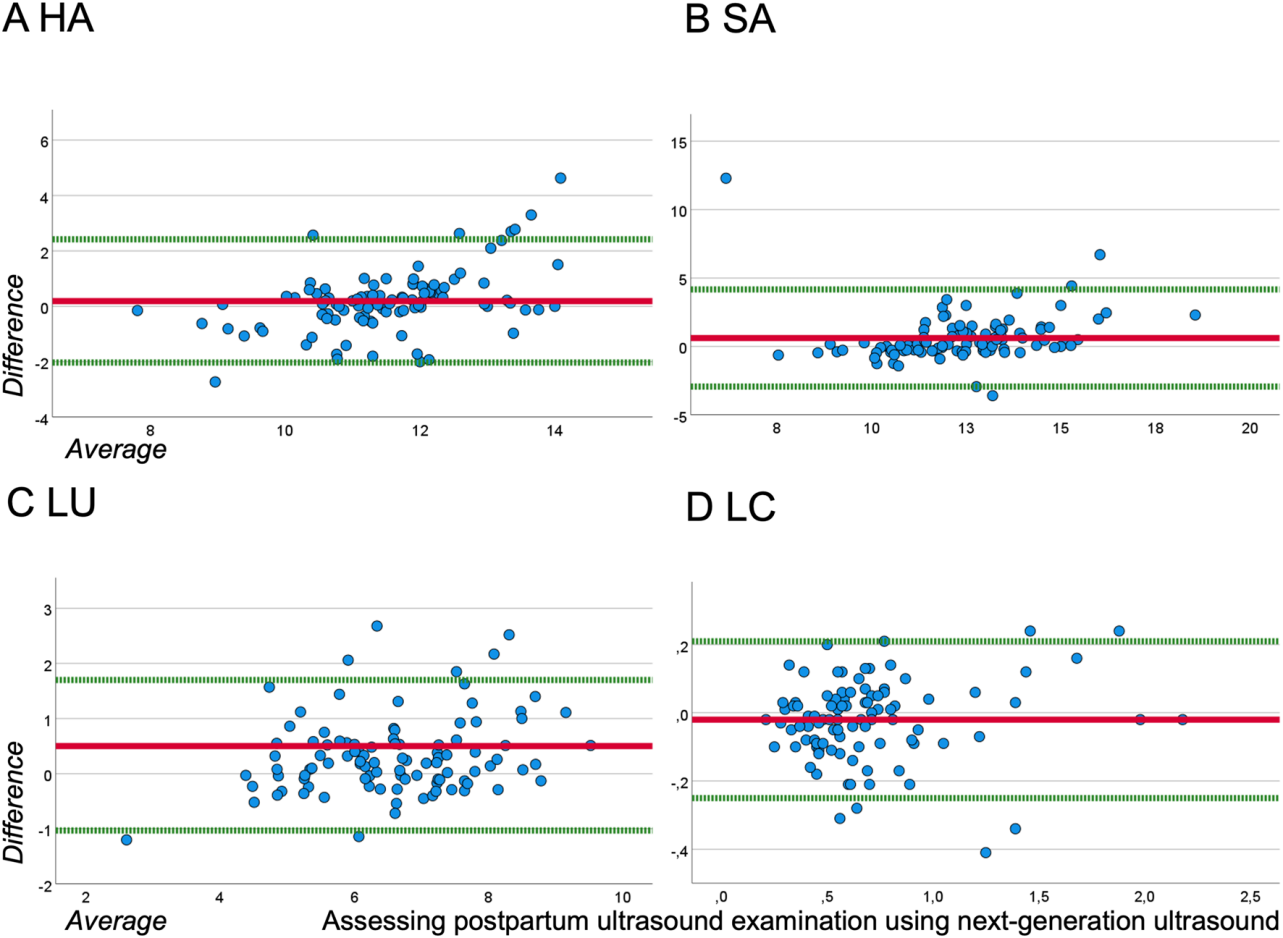
Bland–Altman plot of the uterus and cavum diameters: horizontal axis of the uterus (HU), sagittal axis of the uterus (SU), longitudinal axis of the uterus (LU), and a.-p. axis of the cavity (LC)

For Bland–Altman analysis (Fig. 3 and Table 2), the average difference of HU, SU, LU, and LC was calculated showing an average difference of 0.192, 0.621, 0.331, and − 0.022, respectively. The 95% limits of agreement were between − 2.030 and 2.415 in the case of the HU’s measurement, − 2.933–4.174 cm in the case of the SU’s measurement, − 1.035–1.698 cm in the case of the LU’s measurement, and − 0.254–0.210 in the case of the calculated LC. In the Bland–Altman plot (Fig. 3), the mean difference is close to zero regarding the HU and LU, whereas the mean difference of the SU and LU is around 0.3 and 0.6, displaying larger values measured by SUD than measured by PUD. The MRD of the biometric variables showed values under 8%, except for the LC, where the MRD were 14%.

**Fig. 4:**
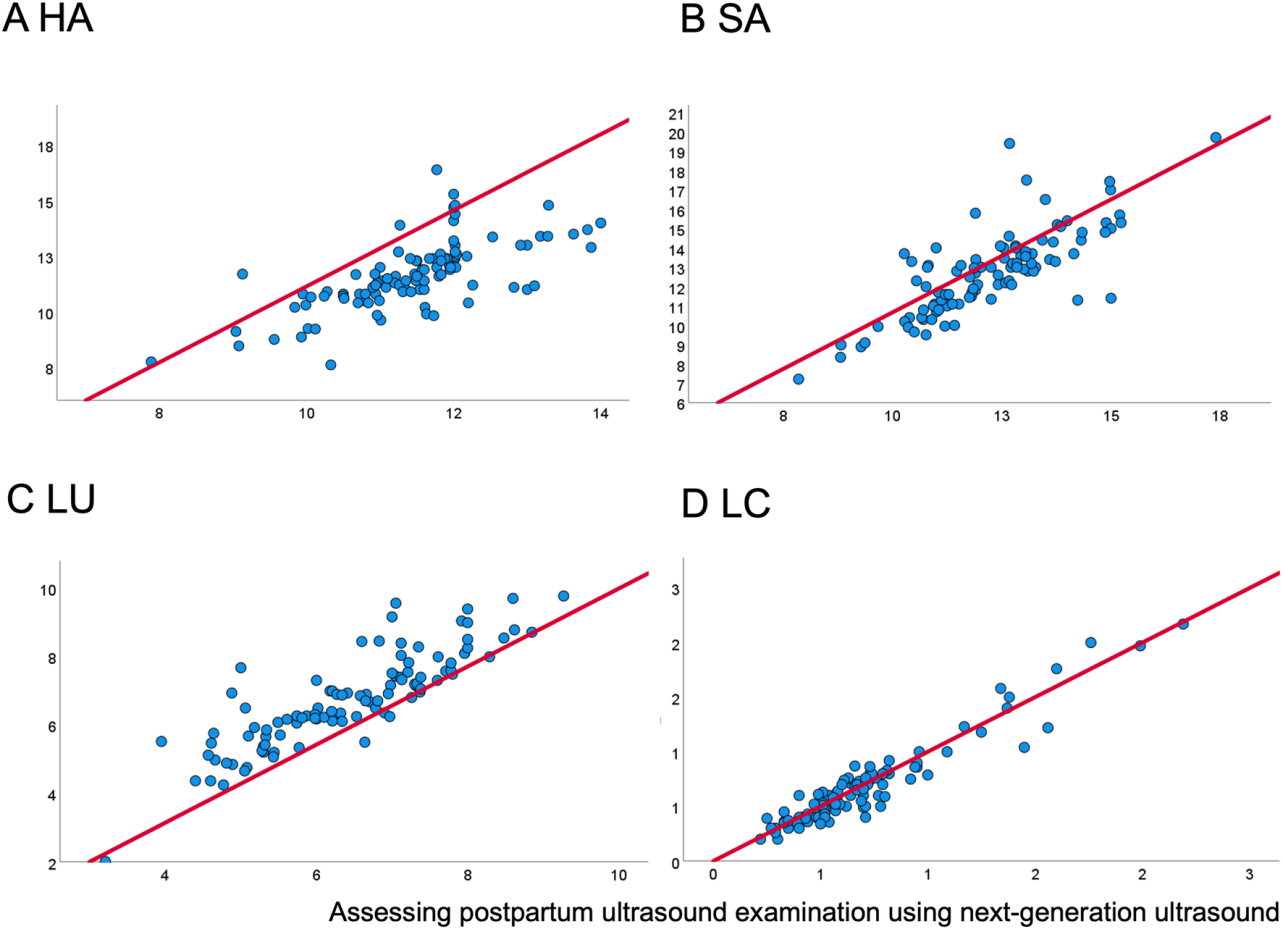
Pearson correlation of the uterus and cavum diameters: horizontal axis of the uterus (HU), sagittal axis of the uterus (SU), longitudinal axis of the uterus (LU), and a.-p. axis of the cavity (LC)

The distribution of all parameters was assessed graphically. No strong deviation from a normal distribution was detected, so that the Pearson correlation coefficient (PCC) could be used. The PCC (Table 3 and Fig. 4) displayed near-perfect correlation for measurements of LU and LC, for which ICCs (Table 4) indicated an excellent agreement as well [20]. The PCC of measurement of the HU and SU also showed a correlation. The ICCs were good for these two variables [20]. The mean diameters of the variables are presented in Table 5.

**Table 3:** Subgroup analysis of the Pearson correlation coefficient (PCC) with a 95% confidence interval using Wald methods between measurements from standard ultrasound device and portable ultrasound device for the horizontal axis of the uterus (HU), sagittal axis of the uterus (SU), longitudinal axis of the uterus (LU), and a.-p. axis of the cavity (LC)

	SVD, *n* = 57	VAVD, *n* = 15	CD, *n* = 28	NW, *n* = 62	OW, *n* = 34	S1PP, *n* = 43	S2PP, *n* = 33	Total, *n* = 100
HU	0.765 (0.590–0.939)	0.705 (0.281–1.130)	0.490 (0.176–0.804)	0.611 (0.406–0.815)	0.832 (0.633–1.032)	0.633 (0.385–0.880)	0.559 (0.255–0.863)	0.690 (0.545–0.835)
SU	0.742 (0.560–0.923)	0.639 (0.179–1.100)	0.812 (0.607–1.041)	0.781 (0.623–0.951)	0.785 (0.562–1.008)	0.758 (0.554–0.981)	0.798 (0.577–1.019)	0.783 (0.661–0.913)
LU	0.861 (0.724–0.999)	0.891 (0.619–1.163)	0.859 (0.675–1.044)	0.873 (0.747–0.999)	0.900 (0.743–1.057)	0.805 (0.615–0.994)	0.908 (0.754–1.061)	0.861 (0.759–0.963)
LC	0.955 (0.876–1.035)	0.957 (0.782–1.131)	0.976 (0.898–1.054)	0.945 (0.860–1.029)	0.965 (0.869–1.060)	0.894 (0.751–1.037)	0.974 (0.892–1.057)	0.951 (0.890–1.013

Assessing postpartum ultrasound examination using next-generation ultrasound

*CD* cesarean delivery, *n* number, *NW* normal weight, *OW* overweight and obesity, *S1PP* scan at the first day postpartum, *S2PP* scan at the second day postpartum, *SVD* spontaneous vaginal delivery, *VAVD* vacuum-assisted vaginal delivery

**Table 4:** Subgroup analysis of the intraclass correlation coefficients (ICC) with 95% confidence interval for agreement between measurements from standard ultrasound device and portable ultrasound device for the horizontal axis of the uterus (HU), sagittal axis of the uterus (SU), longitudinal axis of the uterus (LU), and a.-p. axis of the cavity (LC)

	SVD, *n* = 57	VAVD, *n* = 15	CD, *n* = 28	NW, *n* = 62	OW, *n* = 34	S1PP, *n* = 43	S2PP, *n* = 33	Total, *n* = 100
HU	0.836 (0.722–0.903)	0.826 (0.470–0.942)	0.529 (0.070–0.763)	0.694 (0.494–0.815)	0.898 (0.796–0.949)	0.709 (0.462–0.843)	0.645 (0.281 -0.825)	0.780 (0.673–0.852)
SU	0.797 (0.635–0.885)	0.732 (0.249–0.908)	0.868 (0.733–0.935)	0.831 (0.707–0.901)	0.855 (0.699–0.929)	0.829 (0.681–0.908)	0.821 (0.516–0.923)	0.840 (0.743–0.989)
LU	0.902 (0.752–0.953)	0.945 (0.837–0.981)	0.898 (0.785–0.951)	0.911 (0.798–0.954)	0.931 (0.843–0.967)	0.873 (0.734–0.936)	0.919 (0.801–0.963)	0.905 (0.820–0.945)
LC	0.977 (0.961–0.987)	0.974 (0.924–0.991)	0.987 (0.974–0,994)	0.970 (0.951–0.982)	0.982 (0.964–0.991)	0.942 (0.892–0.969)	0.987 (0.974–0.994)	0.974 (0.962–0.983)

Assessing postpartum ultrasound examination using next-generation ultrasound

*CD* cesarean delivery, *n* number, *NW* normal weight, *OW* overweight and obesity, *S1PP* scan at the first day postpartum, *S2PP* scan at the second day postpartum, *SVD* spontaneous vaginal delivery, *VAVD* vacuum-assisted vaginal delivery

**Table 5:** Mean diameter of the variables measured by the SUD according to the subgroup analysis in cm: horizontal axis of the uterus (HU), sagittal axis of the uterus (SU), longitudinal axis of the uterus (LU), and a.-p. axis of the cavity (LC)

	SVD, *n* = 57	VAVD, *n* = 15	CD, *n* = 28	NW, *n* = 62	OW, *n* = 34	S1PP, *n* = 43	S2PP, *n* = 33	Total, *n* = 100
HU (cm)	11.67	11.05	11.85	11.76	11.34	11.54	11.84	11.63
SU (cm)	13.07	12.35	12.15	12.85	12.56	12.22	13.34	12.70
LU (cm)	6.93	6.08	6.74	6.75	6.75	6.83	6.68	6.75
LC (cm)	0.70	0.68	0.62	0.64	0.74	0.61	0.68	0.67

Assessing postpartum ultrasound examination using next-generation ultrasound

*CD* cesarean delivery, *n* number, *NW* normal weight, *OW* overweight and obesity, *S1PP* scan at the first day postpartum, *S2PP* scan at the second day postpartum, *SVD* spontaneous vaginal delivery, *VAVD* vacuum-assisted vaginal delivery

Regarding the mode of delivery, the PCC and ICC showed similar values in the subgroups: spontaneous vaginal delivery and vacuum extraction for HU, and in all subgroups for LU and LC. The measurement of the HU with the SUD and PUD correlated less in the postpartum ultrasound examination after cesarean delivery in comparison to a postpartum ultrasound examination after spontaneous vaginal delivery and vacuum-assisted vaginal delivery. In the case of SU measurements, the correlation was strongest in the subgroup cesarean delivery, followed by the subgroup spontaneous vaginal delivery and lowest in the subgroup vacuum-assisted vaginal delivery. The ICC of HU was moderate for the measurement after cesarean delivery and good for vaginal delivery and vacuum-assisted vaginal delivery. In regards to the SU, the ICC was moderate in the subgroups vacuum extraction and good in the subgroups spontaneous vaginal delivery and cesarean delivery. An excellent ICC was seen regarding the measurements of the LU after vaginal delivery and vacuum-assisted vaginal delivery, whereas the ICC of the measurement of the LU after cesarean delivery was good. In all three subgroups, the ICC were excellent regarding LC suiting the high correlation as displayed by the PCC.

Upon examining the influence of preconceptional BMI, only the influence of obesity could be analyzed because the number of underweight participants was too low for a statistical analysis. The correlation between the examination with an SUD and PUD was high (over 0.78) except for the measurement of the HU in the subgroup normal weight. The ICC showed similar values in each subgroup for the measured variables of interest except for the HU. The ICC for HU was moderate in the case of patients with normal weight, and good in the case with the overweight/obese. The measurement of the SU revealed similar values in the two subgroups and were good. Excellent ICCs were seen for LU and LC independent of the weight.

Regarding the day of examination, a higher correlation of all measured was detected on the second day in comparison to the first day after the delivery for almost every measured variable except for the HU. The ICCs of the HU were only moderate on the first day, whereas on the second day, the ICC was good. The examinations of the SU on both days und LU on the second day displayed good ICCs. Excellent ICCs were detected by the measurement of the LU at the second day and LC on the first and second days.

All examiners were open to using the PUD again. Their positive and negative feedback is shown in Table 6.

**Table 6:** Feedback of the examiners regarding the new ultrasound device

Positive feedback	Negative feedback
Simple handling	Little screens depending on device (tablet versus smart phone)
Time saving	Empty battery
Low-threshold decision of examination	Acoustic window
Adequate solution	
Examination close to the patient	
Simple maneuverability	
Electricity independence	

Assessing postpartum ultrasound examination using next-generation ultrasound

## Discussion

This study presents the first data regarding the reliability of the new generation of portable ultrasound devices based on silicon chips in postpartum ultrasound examinations. Thereby, the results of the longitudinal diameter of the cavum with the PUD and SUD indicated a high correlation and excellent ICC independent of the analyzed subgroups. The ICC and PCC for the results of the horizontal, sagittal, and longitudinal diameter of the uterus with the PUD and SUD were lower than those of the cavum examination, but correlated nonetheless and showed at least a moderate agreement.

The high score of the PCC and ICC of the cavum’s examination and the accordance of the diagnosis independent on the used device suits to the application area in the delivery room and on the postpartum ward, where the safe and reliable examination of the cavum is vital to secure a definite diagnosis and initiate correct treatment, reducing maternal mortality. This use is supported by the examiner’s feedback which comments a simple handling, and a short preparation time is necessary in the case of emergency. However, the application of the PUD as well as SUD should be used only in the case of clinical pathologies and not for routine examinations, because immediate bedside uterine ultrasound examination performed standardly after manual removal of the placenta is shown to result in unnecessary intervention, without a benefit of the patient outcome [21].

**Fig. 5:**
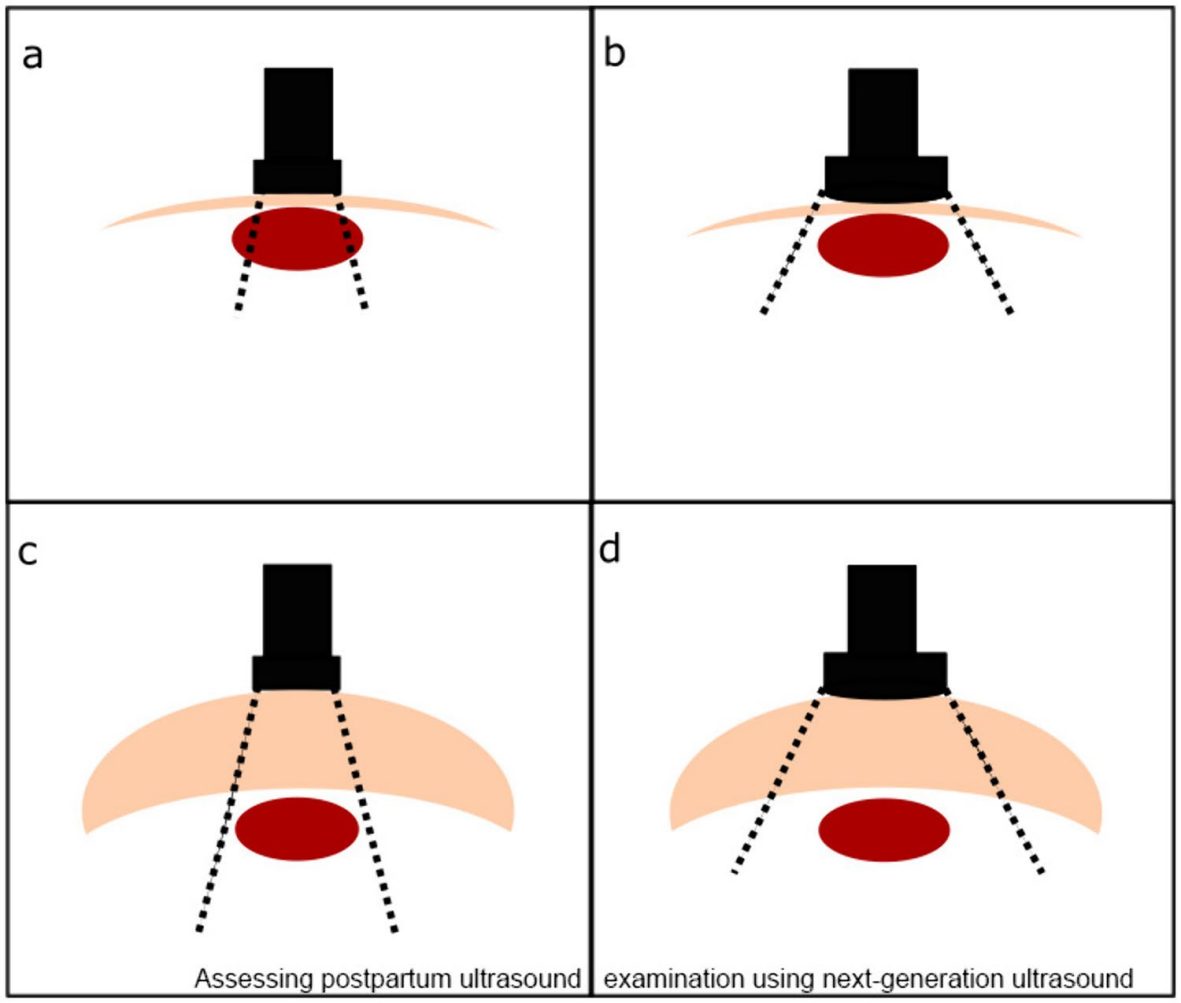
Schematic illustration of a postpartum ultrasound examination in a patient with normal weight (**a**–**b**) or in an overweight patient (**c**–**d**) using either a portable ultrasound device (**a**, **c**) or a standard ultrasound device (**b**,**d**): ultrasound device (black), border of the acoustic window (black dotted line), abdominal wall (beige), transverse section of uterus for measuring the horizontal diameter (red)

Uterine dimensions have been analyzed in depth, so that the physiology of involution is well-known [14, 22]. Based on the individual uterus size in every patient, the involution process is only assessable by repeated examinations at different time points [22]. However, the comparison of the results measured by an SUD and PUD may help to see advantages and disadvantages of the PUD: The moderate ICC and PCC of the examination for horizontal and sagittal diameters coincide with the larger diameter of the HU (mean diameter 11.63 cm) and of the SU (mean SU: 12.7 cm) in comparison with the LU and LC, whereas the good to excellent ICC and PCC could be calculated by measuring the variable of the LU (mean diameter: 6.75 cm) and the LC (mean diameter: 0.67 cm). A modest increasing deviation of the measurement of larger structures is also reported by the fetal biometry at later gestational age [7]. The smaller transducer and, thus, a smaller acoustic window of the PUD (in this device: the smallest diameter is 4 cm) than of the SUD lacks the opportunity to see the whole structure as one in some constellations: The close location of the uterus to the skin in the scan plane for the HU and the linear probe with a flat array of the PUD leads to a too small acoustic window for the short distance between transducer and uterus, which explains the lower correlation in the subgroup normal weight than in the subgroup overweight (larger distance between transducer and uterus) and the different ICC of these subgroups (s. Fig. 5). The measurements of the SU and LU benefit from the anatomic position of the uterus, as the complete SU and LU can be visualized in one image by adjusting the position and angle of the transducer to the abdominal wall and the insonation depth. The different screen size, commented as too small in the case of the PUD, does not relevantly influence the results, as seen by the good PCC and excellent ICC of the measured LU and cavum. The lowest PCC and ICC of all subgroups is seen in the subgroup of cesarean section in regards to the HU measurement, which can be explained by sound cancelation of the scar of the cesarean section. Modified PUD to perform transvaginal applications may solve the problem of noise cancellation [23]. For measurement of the SU, LU, and LC, the position of the transducer was placed  next to  the scar from the cesarean section. In comparison to the ICC and PCC of the variables needed for the estimated fetal weight [7–9, 24] and the bladder volumes, the measurements of the PUD and SUD have a wider range, which may be caused by the less defined scan planes for the uterus in comparison to other scan planes such as the head circumference.

## Limitations

Several limitations are inherent in its design and scope that merit consideration: Primarily, as single-center study, the unique environment and patient demographic of a tertiary-level university hospital does not reflect diverse settings of others healthcare systems. Furthermore, the exclusion of emergent cases allows a safe realization of the study, but simultaneously omits a highly relevant group of patients which could greatly benefit from PUD in the future. In a next step, the use of PUD in diagnosing intrauterine pathology should be tested in cases with clinical pathologies. The power of 100 patients and the study design limit the generalizability of the results to broader clinical practices and lead to unbalanced subgroups, which skew the analysis of the impact of these factors on the performance and reliability of PUD compared to SUD. The performing physicians’ different levels of ultrasound experience, ranging from several years to decades of practice, may lead to discrepancies in ultrasound examination skills and interpretation and, thus, to inconsistency. These variables may affect the comparative analysis of SUD and PUD, potentially leading to bias in the accuracy and reliability of measurements.

The study was performed with one portable ultrasound system with a non-piezo, chip-based technology. This new technology is under constant development and almost every ultrasound manufacturer has now introduced a model on the market. Thereby, each manufacturer has its own platforms and apps with individual technical specifications. These different technical specifications, cost-effectiveness, and the potential for enhancing access in underserved communities were not explored in our study, despite their crucial factor for an integration of PUD into general obstetric care.

Long-term impacts, clinical outcomes, and decision-making in patient management have to be analyzed in a follow-up study.

## Conclusion

This pilot study regarding the accuracy and reliability of non-piezo portable ultrasound devices in a postpartum ultrasound examination offers promising results for an application in postpartum care to improve point-of-care ultrasound and support rapid diagnosis-making in the case of an emergency. The assessment of the cavum with a PUD leads to the same diagnosis as with a SUD and to a near-perfect correlation and agreement analyzed by the PCC and ICC. The diameters of the uterus as further comparative example pinpoint weaknesses of the PUD, specifically the small ultrasound window, but this weakness lacks clinical relevance, especially in emergency situations. Our findings underscore the high reliability of PUDs for basic obstetric evaluations, as reported in other studies, and support their safe integration into daily clinical practice. This technology has the potential to enhance the flexibility and immediacy of postnatal care, making real-time assessments more accessible in a variety of settings.

## Data Availability

The data that support the findings of this study can be requested by the authors. The access is subject to approval and restricted due to legal and ethical reasons.
